# Bioactivity-Guided Screening of Wound-Healing Active Constituents from American Cockroach (*Periplaneta americana*)

**DOI:** 10.3390/molecules23010101

**Published:** 2018-01-20

**Authors:** Juan-Juan Zhu, Shun Yao, Xin Guo, Bi-Song Yue, Xiu-Ying Ma, Jing Li

**Affiliations:** 1 Key Laboratory of Bio-Resource and Eco-Environment of Ministry of Education, College of Life Sciences, Sichuan University, Chengdu 610065, China; zjj20045@163.com (J.-J.Z.); guoxinshangan@sohu.com (X.G.); bsyue@scu.edu.cn (B.-S.Y.); 2 Department of Pharmaceutical and Biological Engineering, School of Chemical Engineering, Sichuan University, Chengdu 610065, China; cusack@scu.edu.cn; 3 Sichuan Gooddoctor-Panxi Pharmaceutical Co., Ltd., Xichang 615000, China; mxy0701@hys.cn

**Keywords:** *Periplaneta americana*, wound-healing activity, active constituents, bioactivity-guided screening

## Abstract

Ethanol extract (EE) from *Periplaneta americana* (PA) is the main ingredient of Kangfuxin, which is a popular traditional chinese medicine (TCM) and has long been used for the clinical treatment of burns, wounds and ulcers. We compared the wound-healing activities of three extracts of PA using cutaneous wound-healing in mice as the bioactivity model. These three extracts were EE, total polysaccharide and total protein. We also tracked bioactive fractions in the EE by organic reagent extraction, column chromatography and HPLC. Seven compounds were successfully identified from the water elution fraction of the EE of PA using UPLC-MS. Among these compounds, four compounds (**P2**, **P3**, **P4**, **P5(1)**) were first reported in PA. Some of these compounds have been previously reported to have various pharmacological activities that could contribute to the high wound-healing activity of PA.

## 1. Introduction

Wound healing is a complex biological process including three classic stages: inflammation, new tissue formation and remodeling [1]. Any aberrancies at each stage can lead to delayed wound healing. Many synthetic drugs and artificial skins are used for wound healing, but they are expensive and readily cause allergic reactions in sensitive people [2,3,4]. Thus, there is a real need for an alternative to synthetic wound-healing products. Natural products are the most reliable and successful sources of drug leads. For example, insects have been increasingly used as effective bioactive products and as they are one of the most diverse taxa of living organisms can provide a considerable resource of potential alternatives [5]. Therefore, the potential for insect constituents, such as cockroaches, to enhance natural wound healing is a particularly important research avenue. 

*Periplaneta americana* (PA), the American cockroach, has the largest body size in the family Blattidae. It is also one of the most famous sanitation-related insects with strong vitality and successful reproduction [6]. PA is widely distributed in subtropical and tropical regions across the world [7]. Many previous PA studies have focused on the infestation of human dwellings and the strong ability to transmit pathogenic fungi [8]. However, other physiological and pharmacological studies have demonstrated that PA constituents have favorable tissue-repairing [9], antibacterial [10], antitumor [11] and immunity-enhancing activities [12]. Additionally, this insect has been used in traditional chinese medicine (TCM) as an important biomedical component for the treatment of TCM syndromes such as blood stasis, ulcers, burns and wounds for hundreds of years [13]. Consequently, the formulations of many TCM preparations, such as Kangfuxin Liquid, Ganlong Capsule, Xinmailong Injection, and Xiaozheng Yigan Tablet, include ingredients from PA [14]. Among them, Kangfuxin is a liquid preparation that has been used to treat different skin or mucosa injuries in China for more than 40 years [15]. According to the previous component analysis on PA, the reported chemical components of the cockroach mainly include pheromones, amino acids, insect neuropeptides, adipokinetic hormones and dihydroisocoumarins [16]. However, the effective ingredients in PA that can promote the wound-healing process remain largely unknown. The purpose of this study is to investigate related active chemical components that demonstrate efficacy in wound healing. In the present study, we systematically screened the bioactive compounds or fractions with wound-healing activities in PA. The ethanol extract (EE), total polysaccharide and total protein of PA were used to compare their wound-healing activities on the basis of the cutaneous injured mice model. Then, we performed systematic separation and analysis on the EE of PA, including organic reagent extraction, macroporous resin column chromatography, High Performance Liquid Chromatography (HPLC) and Ultra Performance Liquid Chromatography-Mass Spectrometry (UPLC-MS).

## 2. Results and Discussion

### 2.1. Comparison of Wound-Healing Activities of Different PA Extracts

**Figure 1:**
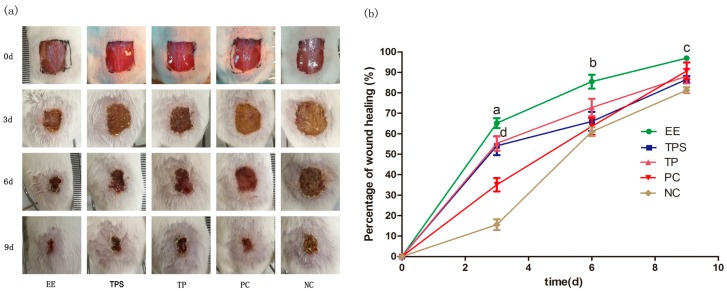
Wound-healing assay of mice treated with ethanol extract (EE), total polysaccharide (TPS), total protein (TP), Jingwanhong (positive control—PC) and pure water (negative control—NC) at 0, 3, 6 and 9 days. (**a**) Photographs of dorsal excisional skin wounds on different days. Day 0 pictures were taken immediately after wounding; (**b**) Data are given as mean ± standard deviation (SD) for three mice in each group. Statistical analysis of wound area used one-way ANOVA followed by Tukey’s Honestly Significant Difference (HSD) post hoc test. The characters indicate statistically significant differences (*p* < 0.05): a, b and c: EE compared with other four groups on days 3, 6 and 9; d: between TP/TPS and NC on day 3.

The wound-healing activities of the EE, polysaccharide (TPS) and total protein (TP) of PA are shown in Figure 1a. Compared to other PA extracts, the EE-treated wounds became dry, and scabs began to form on the first day of treatment, likely as a result of the adjacent skin cells interacting with the scab to promote wound healing [17]. The sizes of the wounds decreased significantly at the third and sixth day in the EE-treated group. Scabs of wounds treated with EE started to drop off on the ninth day, and the granulation tissue below the wound grew swiftly and showed a pinkish color. However, pus and blood were observed in the wounds of the TPS and TP group mice even after the sixth day. The results indicated that EE-treated wounds scabbed faster, and had smaller wound sizes and less swelling than those treated with TPS, TP and Jingwanhong (i.e., positive control—PC).

We found that the healing rates of the EE-treated group were the highest at each time interval compared to the TPS, TP and negative control (NC) groups (Figure 1b). Moreover, the healing rate of the EE-treated group significantly increased (*p* < 0.05) to 65% at the third day, whereas the healing rate was only 16% in the NC group. We noted that the percentage increases in wound healing slowed at the sixth day and ninth day, which may be related with the formation of scabs. Previous studies have suggested that the scabs could slow the wound-healing process and prevent the migration of epithelium cells on the surface of the wound exudates [18,19]. We also noted that Jingwanhong-treated wounds were slow to heal at day 3 but exhibited a similarly high healing rate (91%) to the EE-treated group at the ninth day. These results suggested that different wound-healing active compounds might exist in the EE of PA and Jingwanhong. In addition, the TPS- and TP-treated groups exhibited significantly high healing rates only at the third day as compared to both of the control groups (*p* < 0.05). 

### 2.2. Separation by Organic Reagent Extraction and Macroporous Resin Column Chromatography

EE was separated by organic reagent extraction to obtain chloroform fraction Fr.A (0.3169 g), ethyl acetate fraction Fr.B (0.5208 g), *n*-butanol fraction Fr.C (4.3195 g) and water fraction Fr.D (55.5534 g). The yield rates and characteristics of the four fractions with organic reagent extraction are shown in Table 1. It was found that the yield rates increased with the polarity of the extracted organic reagents, with the lowest yield in the chloroform fraction (Fr.A) and the highest yield in the water fraction (Fr.D) (11%). The bioactivity analysis also indicated that Fr.D had a better wound-healing activity than the other three fractions. When Fr.D was applied to the cutaneous wounded mice, the wound-healing rate reached 93.6% at the ninth day, whereas it reached only 9.4% when Fr.A was applied (Table 1). The results indicated that highly polar constituents predominant in the EE may contribute to the wound-healing activity.

Fr.D (30 g) was then fractionated on macroporous resin column with different gradients of ethanol generating six subfractions of water elution fraction Fr.D1 (23.184 g), 10% ethanol fraction Fr.D2 (1.604 g), 30% ethanol fraction Fr.D3 (1.87 g), 50% ethanol fraction Fr.D4 (0.404 g), 70% ethanol fraction Fr.D5 (0.02 g) and 90% ethanol fraction Fr.D6 (0.007 g). The Fr.D1 had the highest yield of 77.3%, and it showed the highest wound-healing activity compared to the other subfractions. When the bioactivities of Fr.D and Fr.D1 were compared (Table 2), Fr.D1-treated wounds showed a nearly 100% healing rate at the ninth day, while the healing rate of the Fr.D-treated group was around 92%. Additionally, Fr.D1-treated mice showed better recovery symptoms, including a light pink dermis without any crusts, prominent wound contraction and re-epithelialization, and active granulation tissue, compared to Fr.D. The results suggested that wound-healing active compounds in EE had been concentrated after column chromatography.

### 2.3. UPLC-MS Analysis of Wound-Healing Activity Fraction

**Figure 2:**
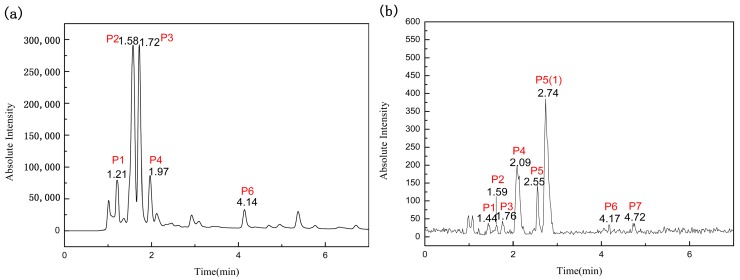
UPLC chromatogram (five peaks) (**a**) and total ion chromatogram (seven peaks) (**b**) at 254 nm (here are shown only intercept chromatograms for the first 7 min because there was no peak after then).

The UPLC chromatogram of Fr.D1 (Figure 2a) illustrated five peaks (**P1**–**P4** and **P6**), which were in agreement with those shown in the total ion chromatogram (Figure 2b). We noted that three additional peaks (**P5**, **P5(1)** and **P7**) were detected in the total ion chromatogram but were absent in the UPLC chromatogram. This result suggested that **P5**, **P5(1)** and **P7** were highly likely to be compounds with a weak absorption at 254 nm. In addition to the five distinct peaks, three peaks with low response values at retention times of 2.12, 2.92 and 5.37 min were detected in UPLC. They could not correspond to those of the total ion current because of improper mass spectrometry conditions and ion mode. The corresponding mass chromatograms of seven compounds are shown in Supplemental Material (Figure S1).

By integrating UPLC with HRMS (MS1), we successfully identified seven compounds in the Fr.D1 corresponding to peaks **P1**–**P7**. The molecular weight, structure and molecular formula of the compounds were searched for in the SCIfinder database and relevant literature (Table 3). Compounds **P5** and **P5(1)** in Figure 2b were highly likely to be isomers according to the mass spectrometry data. Here we only identified compound **P5(1)** with a high content. Three of the seven compounds have been previously reported (compound **1**: cyclo-(l-Val-l-Pro); compound **6**: 7-hydroxycotadecanoic acid; compound **7**: (*S*)-2,3-dihydroxypropyl hexadecanoic acid ester). Whereas the other four compounds (compound **2**: 2-(4′-methyl-3′-pentene)-6-hydroxymethyl-10-methyl-12-hydroxyl-(2,6,10)-triendodecanic acid; compound **3**: arbutin; compound **4**: 4-benzyloxy-3-methoxybenzoic acid; compound **5(1)**: (*E*)-3-hexenyl-β-d-glucopyranoside) were first reported in PA.

Compounds **3** and **5(1)** are glycosides that have not been previously reported in PA. Compound **3** (arbutin) displayed a [M + H]^+^ ion at *m*/*z* 273.0745 and a molecular formula C_12_H_16_O_7_ (molecular weight: 272.0745 Da). The peak area of compound **3** was high in the UPLC chromatogram compared to other compounds, which indicated a high content of arbutin in Fr.D1. As a naturally occurring glycoside, arbutin is known for various pharmacological activities, particularly the anti-inflammation and anti-ulcer activities [26]. Arbutin has been found in several plants, such as *Vaccinium vitis idaeae* [27], *Pyrus bretschneideri* [28] and *Onobrychis viciaefolia* Scop [22]. However, it has not been found in PA or other insects. Three different periplanosides have been previously isolated from PA that share similar structures with arbutin [16]. One of the three periplanosides stimulates collagen production that is closely related with skin and ulcer repair [16]. Considering the similar structure to other periplanosides with high pharmacological activities and the high content in Fr.D1, arbutin was probably an important component contributing to the observed wound-healing activity. Compound **5(1)** showed a [M + H]^+^ ion at *m*/*z* 263.1840 and the molecular formula C_12_H_22_O_6_. It was originally extracted from *Hylomecon vernalis* Maxim with little cytotoxicity against some human tumor cell lines [24] and was first found in PA. 

Compounds **2** (a diterpenoid) and **4** (a phenolic acid) were identified from the Fr.D1 of PA for the first time. Compound **2** showed a [M + H]^+^ ion at *m*/*z* 337.0458, and the molecular formula was C_20_H_32_O_4_. It was originally isolated from the aqueous extract of *Smallanthus sonchifolius,* and the crude extract of this plant has been found to have antidiabetic activity [21]. Research on diterpenoids extracted from various plants has confirmed several biological activities, for example, anti-inflammation [29], antimicrobial [30], antitumor [31] and antioxidant [32] activities. Compound **4** (C_15_H_14_O_4_) had an elemental composition of the [M + H]^+^ cation at *m*/*z* 259.0785 and was reported with high antioxidant activities [23]. Antioxidants can eliminate free radicals that are involved in inflammatory and cardiovascular diseases [33]. 

Compound **1** cyclo-(l-Val-l-Pro) is a cyclic peptide, which was 1 of 10 reported cyclic peptides initially separated from the ethyl acetate fraction of the PA EE [20]. Previous research has suggested that many natural cyclic peptides have more stable structures than linear peptides and have diverse biological activities such as antitumor, immune-regulatory and antimicrobial activities [34]. Therefore, this compound may have played a role in the process of the cutaneous wound repair of the studied mice.

Compounds **6** and **7** are long-chain fatty acids that were previously isolated from the ethyl acetate extract and the chloroform extract of PA [7,25], respectively. Many types of fatty acids have been identified in PA, including long-chain polyunsaturated fatty acids, benzoic acid and squalene, some of which showed biological activities, such as anti-inflammation, antibacterial and antitumor activities [35,36,37]. 

Considering that wound healing is a dynamic and extremely complicated process [1], synergistic effects from different bioactive compounds likely contribute to the high wound-healing activity of Fr.D1. Further studies are expected to elucidate the wound-healing compounds in PA.

## 3. Materials and Methods

### 3.1. Reagents and Experimental Animals

Chloroform, ether, ethanol, ethyl acetate, and *n*-butanol for separation were of analytical grade. Methanol and acetic acid for UPLC were of chromatographic grade. D101 macroporous resin was purchased from the Lingyun Instrumental Factory (Chengdu, China). Jingwanhong (No. 211991), a traditional Chinese medicine for burns, scalds and wound healing [38] and produced from the Pharmaceutical Industry Limited Company (Tianjin, China), was used as the PC. Male Specific pathogen Free (SPF) Kunming mice weighing 18–22 grams were purchased from the Chengdu Dashuo Experimental Animal Limited Company (Chengdu, China).

### 3.2. Preparation of Three PA Extracts

PA was obtained from the Good Agriculture Practice (GAP) breeding base, Sichuan, China. The powdered dried PA (500 g) was extracted with 90% ethanol (3 L) twice at 80 °C. After solvent evaporation, the EE was recovered and the yield rate in the PA powder was 21%. The EE was dissolved in distilled water (100 mg/mL) for bioactivity assays. TPS and TP of PA were both supplied by the molecular biology laboratory of the College of Life Science, Sichuan University. TPS was extracted from PA powder using 0.02 mol/L of NaOH (70 °C, 2 h) with a yield rate of 17.0% in PA powder, whereas TP was extracted by tris-HCl (35 °C, 6 h, pH 6.0) with a yield rate of 16.2%. TPS and TP were dissolved in distilled water at concentrations of 100 mg/mL for the bioactivity assays. EE showed the highest activity among the three PA extracts and was then chosen for the following isolation.

### 3.3. Organic Reagent Extraction and Macroporous Resin Column Chromatography Isolation

The organic reagent extraction for EE was based on the method of Yuanhui Li [39]. EE (500 mL) was sequentially extracted three times (500 mL × 3) with chloroform, ethyl acetate and *n*-butanol. All layers were combined and concentrated under vacuum to obtain the chloroform fraction (Fr.A), ethyl acetate fraction (Fr.B), *n*-butanol fraction (Fr.C) and water fraction D (Fr.D). All of the samples were maintained at −20 °C until use. Among these fractions, Fr.D showed the highest bioactivity and was subsequently used for further macroporous resin column chromatography. 

**Figure 3:**
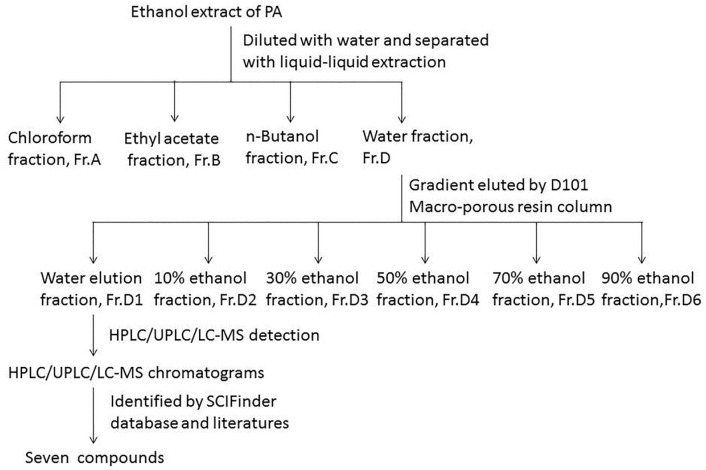
Separation and analysis procedure of ethanol extract from *Periplaneta americana* (PA).

D101-type resin (300 g) was weighed and soaked in ethanol for 24 h, and then a resin column (4 cm × 50 cm) was packed by a wet method [40]. Subsequently, absolute ethanol and distilled water were used to wash the resin in turn until there was nearly no turbidity in elution. Then 30 g of Fr.D was loaded after being dissolved in distilled water. After the sample was completely absorbed, the resin was successively eluted with an increasing gradient ethanol at a flow rate of 3 mL/min. Six fractions were yielded, Fr.D1, Fr.D2, Fr.D3, Fr.D4, Fr.D5 and Fr.D6. All fractions were condensed and weighed. All samples were maintained at −20 °C for use. The Fr.D1 possessed a better activity than Fr.D and was followed by analytical HPLC. The scheme of extraction and isolation procedure is shown in Figure 3.

### 3.4. UPLC-MS Identification and Analysis

Chromatographic separation was performed on a Waters Acquity UPLC system equipped with a C18 column (2.1 × 100 mm, 1.8 μm). The mobile phase consisted of solvents A (0.1% formic acid in water) and B (methanol). The injection volume was 5 μL. A flow rate of 0.2 mL/min for a total run time of 15 min with a linear gradient was used as follows: 0–2 min, 3–5% B; 2–5 min, 5–10% B; 5–10 min, 10–20% B; 10–15 min, 20–30% B. The UPLC-MS analysis was carried out using a Q-TOF Premier system with an electrospray ionization (ESI) source in the positive ion mode (Quattro Premier XE Mass Spectrometer, Waters, USA). The main parameters of UPLC-MS were as follows: capillary voltage at 2.8 eV, extraction cone at 3.5 V, cone gas flow at 25 L/h, source temperature at 90 °C, collision energy at 5 eV, sampling cone at 40 V, desolvation temperature at 200 °C, and desolvation gas flow at 300 L/h. The chromatograms and mass spectral data were compared with that of the reference data, and the molecular weight, formula and structure of the compounds identified were determined through the SCIfinder database.

### 3.5. Assay of Wound-Healing Activity

The wound-healing activities of EE, TPS and TP of the PA extracts were compared. Next, EE was further divided into Fr.A, Fr.B, Fr.C and Fr.D, and the wound-healing activities of each fraction were measured. The activities of Fr.D and Fr.D1 isolated from Fr.D were also investigated.

#### 3.5.1. Animal Modeling of Cutaneous Excision Wound and Administration

All male SPF mice were housed at room temperature and fed with water and food freely for one week to acclimatize to laboratory conditions before the experiment. All experimental procedures were in accordance with the “Guidelines for the Care and Use of Animals in Research” of the Institute of Zoology, Chinese Academy of Sciences. The mice were randomly divided into experimental groups, a PC group and a NC group, with three animals in each group.

All of the mice were inhibited from drinking water the night before the operation. The hair on the dorsal side of the mice was removed with 8% sodium sulfide, and then the mice were put back in cages after cleaning the exposed skin area with warm water. On the day of the surgery, the mice were anesthetized by an intraperitoneal injection of 10% chloral hydrate (0.003 mL/g), and a 1.4 cm × 1.4 cm symmetrical square was outlined along the dorsal middle line. The skin was sterilized using 75% alcohol, and a wound was created by excision of the superficial fascia according to the outlined square [41,42]. The mice were then housed individually in cages.

The mice of the experimental groups and NC group were treated with 30 μL of extracts and water by dripping slowly after 2 h of modeling, respectively, while Jingwanhong ointment was evenly applied onto the wound area with medical cotton stickers of the PC group mice. The wounds were left open after administering either the treatment or control, and the mice were put back into clean cages. The administration for each group was conducted once a day for 9 days. In the process of modeling and administration, the mice all showed a good mental state and activity.

#### 3.5.2. Evaluation of Wound-Healing Activity and Statistical Analysis

The cutaneous wounds of mice were photographed, and the wound areas were measured by tracing the margins after the injury (day 0), on the third, sixth and ninth days. The percentage of wound healing was calculated with the following formula [41]:

Percentage of wound healing (%) = (original area − unhealed area)/original area × 100

The wound-healing percentage was analyzed by one-way ANOVA followed by Tukey’s HSD post hoc test using statistical software package (SPSS) and GraphPad Prism software (version 5.0, GraphPad Software Inc., San Diego, CA, USA). All results are displayed as mean ± standard deviation (SD), and *p* < 0.05 was considered as statistically significant.

## 4. Conclusions

Through the combination of traditional separation strategies with bioactivity tracking, we successfully found that the water elution fraction (Fr.D1) of EE of PA had the highest healing activity. Seven compounds (including cyclopeptide, diterpenoid, phenolic acid, fatty acids and glycosides) were identified from this fraction with the UPLC-MS method, among which the diterpenoid, one phenolic acid and the two glycosides were first reported in PA. Most of the identified compounds have been reported to have antibacterium, anti-inflammation or enhancing immunity activities that were highly likely to be associated with wound repair for mice. The study is expected to lay the foundation for further research and utilization of PA.

## Figures and Tables

**Table 1:** The yield, color and healing rate of different solvent extracts (Fr.A: chloroform fraction; Fr.B: ethyl acetate fraction; Fr.C: *n*-butanol fraction; Fr.D: water fraction D) of 500 mL of *Periplaneta Americana* (PA) ethanol extract.

Extract	Yield (%) *	Color of Concentrate	Healing Rate (Mean ± SD)% **
Fr.A	0.063	Red brown	9.4 ± 1.4
Fr.B	0.104	Red brown	75.4 ± 3.7
Fr.C	0.864	Tan	61.5 ± 5.6
Fr.D	11.111	Black brown	93.6 ± 4.7

* The yield rate means the proportion of various solvent extracts in the PA powder. ** The healing rate of various extracts on day 9 (pure water—negative control group: 71.3 ± 3.4%; Jingwanhong—positive control group: 92.3 ± 5.8%). SD: standard deviation.

**Table 2:** Comparison of water fraction Fr.D and water elution fraction Fr.D1 on day 9 in wound-healing (PC: positive control group; NC: negative control group) activities.

Fraction	Color *	Healing Rate (Mean ± SD)% *
Fr.D	Red	92.3 ± 2.8
Fr.D1	Light pink	99.5 ± 0.8
PC	Light pink	92.6 ± 2.3
NC	Reddish-brown	71.3 ± 3.4

* The color and healing rate of wounds on day 9. SD: standard deviation.

**Table 3:** Compounds identified from the water elution fraction (Fr.D1) of *Periplaneta Americana* (M.F.: molecular formula).

No.	Ion Peak	*m*/*z*	Compound	M.F.	Structure	Reference
**P1**	[M + K]^+^	235.1451	Cyclo-(l-Val-l-Pro)	C_10_H_16_N_2_O_2_	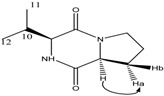	[20]
**P2**	[M + H]^+^	337.0458	2-(4′-Methyl-3′-pentene)-6-hydroxymethyl-10-methyl-12-hydroxyl-(2,6,10)-triendodecanic acid	C_20_H_32_O_4_	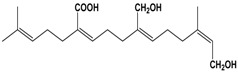	[21]
**P3**	[M + H]^+^	273.0745	Arbutin	C_12_H_16_O_7_	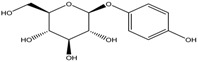	[22]
**P4**	[M + H]^+^	259.0785	4-Benzyloxy-3-methoxybenzoic acid	C_15_H_14_O_4_	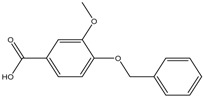	[23]
**P5(1)**	[M + H]^+^	263.1840	(*E*)-3-Hexenyl-β-d-glucopyranoside	C_12_H_22_O_6_	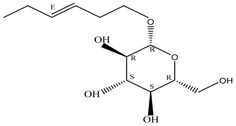	[24]
**P6**	[M + K]^+^	339.0539	7-Hydroxycotadeca-noic acid	C_18_H_36_O_3_	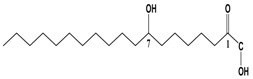	[25]
**P7**	[M + Na]^+^	331.1576	(*S*)-2,3-Dihydroxypropyl hexadecanoic acid ester	C_19_H_38_O_4_	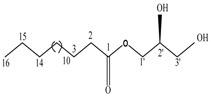	[7]

## Supplementary Materials

The supplementary materials are available online.
