# Supplementary material for: Bioactivity-Guided Screening of Wound-Healing Active Constituents from American Cockroach (Periplaneta americana)
Source: Molecules. 2018 Jan 20;23(1):101. doi: 10.3390/molecules23010101 (PMC6017267; doi:10.3390/molecules23010101)
Supplement: Supplementary file 1 [file molecules-23-00101-s001.pdf]

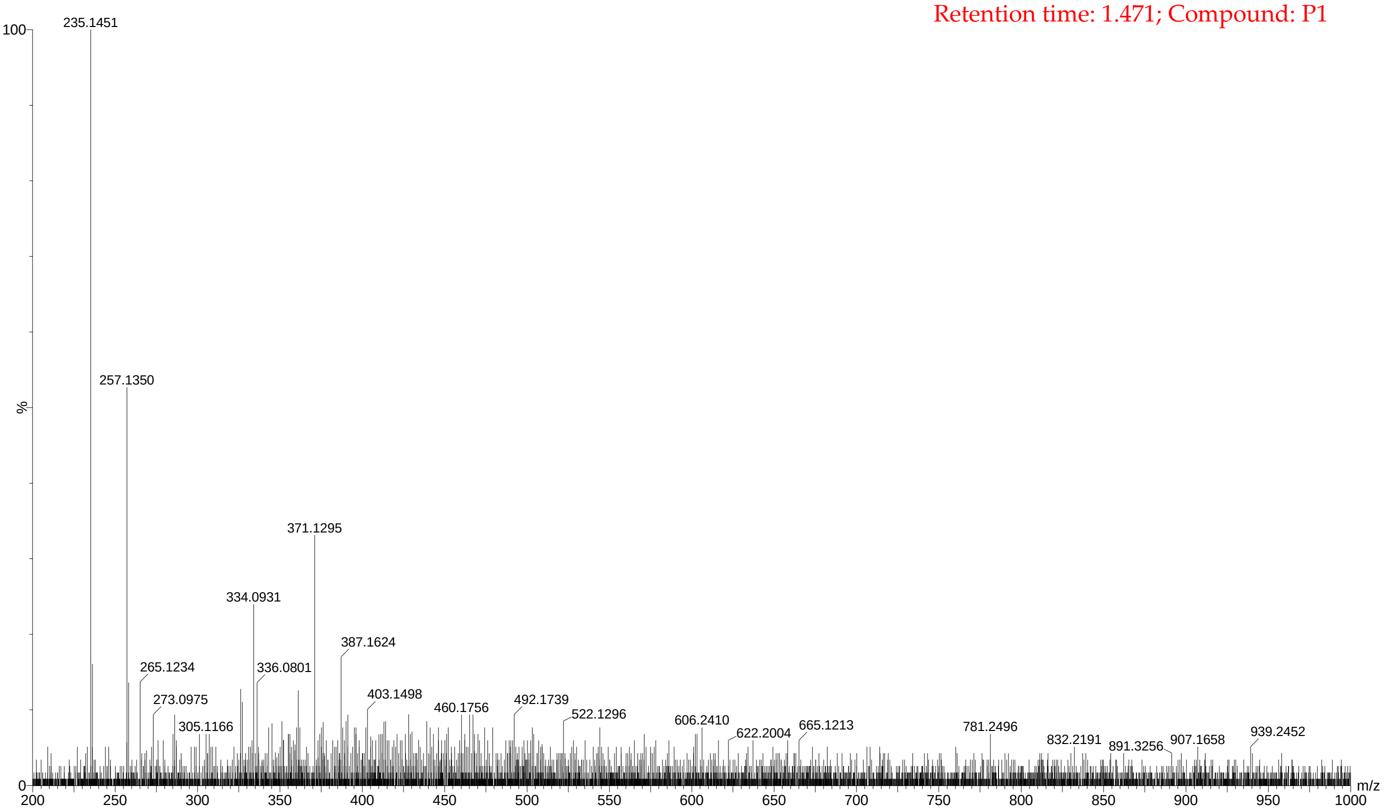
Retention time: 1.471; Compound: P1
235.1451
257.1350
371.1295
334.0931
387.1624
265.1234
336.0801
273.0975
403.1498
460.1756
492.1739
305.1166
522.1296
606.2410
622.2004
665.1213
781.2496
832.2191
891.3256
907.1658
939.2452
%
100
0
m/z
200
250
300
350
400
450
500
550
600
650
700
750
800
850
900
950
1000

(**a**)

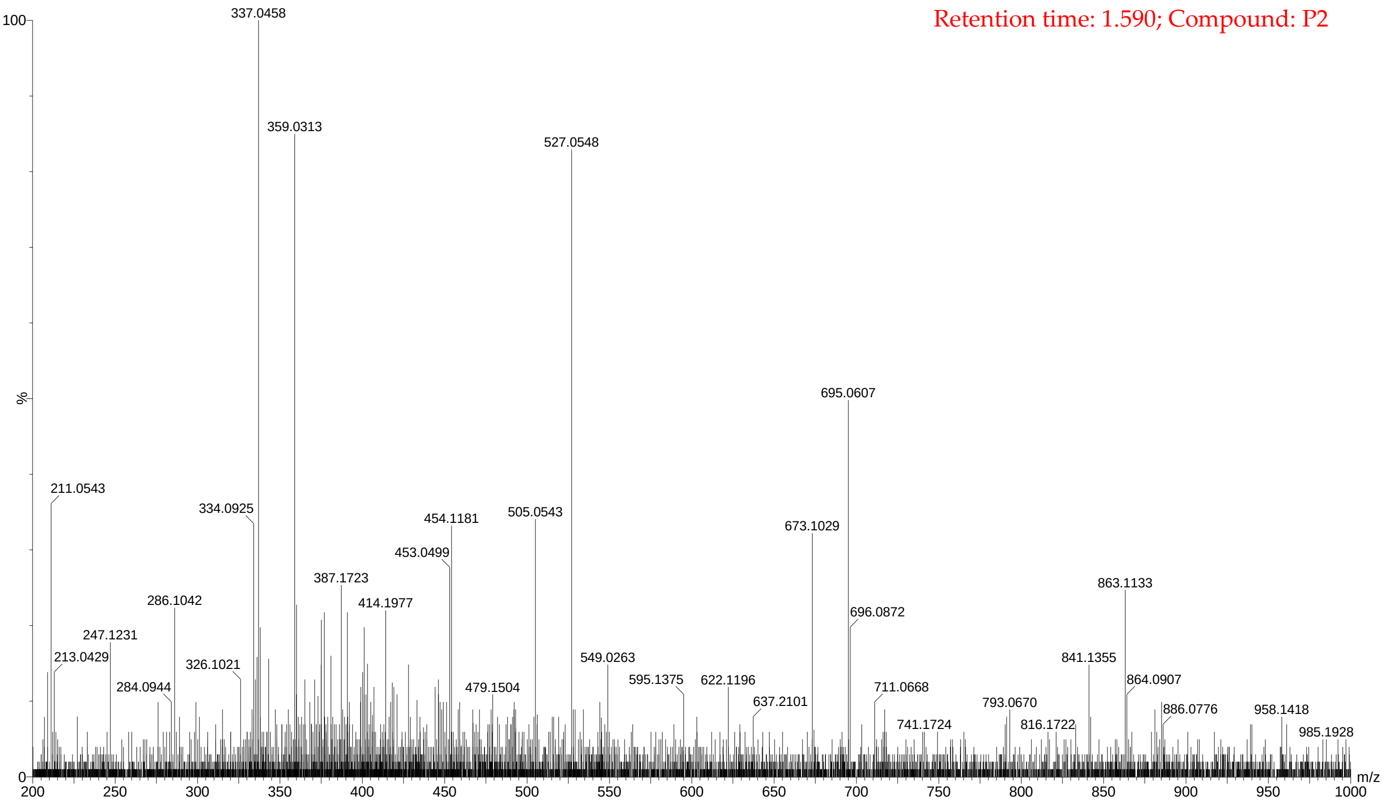
Retention time: 1.590; Compound: P2
337.0458
359.0313
527.0548
695.0607
211.0543
334.0925
454.1181
505.0543
673.1029
453.0499
387.1723
863.1133
286.1042
414.1977
696.0872
247.1231
213.0429
326.1021
549.0263
841.1355
284.0944
479.1504
595.1375
622.1196
864.0907
711.0668
637.2101
793.0670
886.0776
958.1418
741.1724
816.1722
985.1928
%
100
0
m/z
200
250
300
350
400
450
500
550
600
650
700
750
800
850
900
950
1000

(**b**)

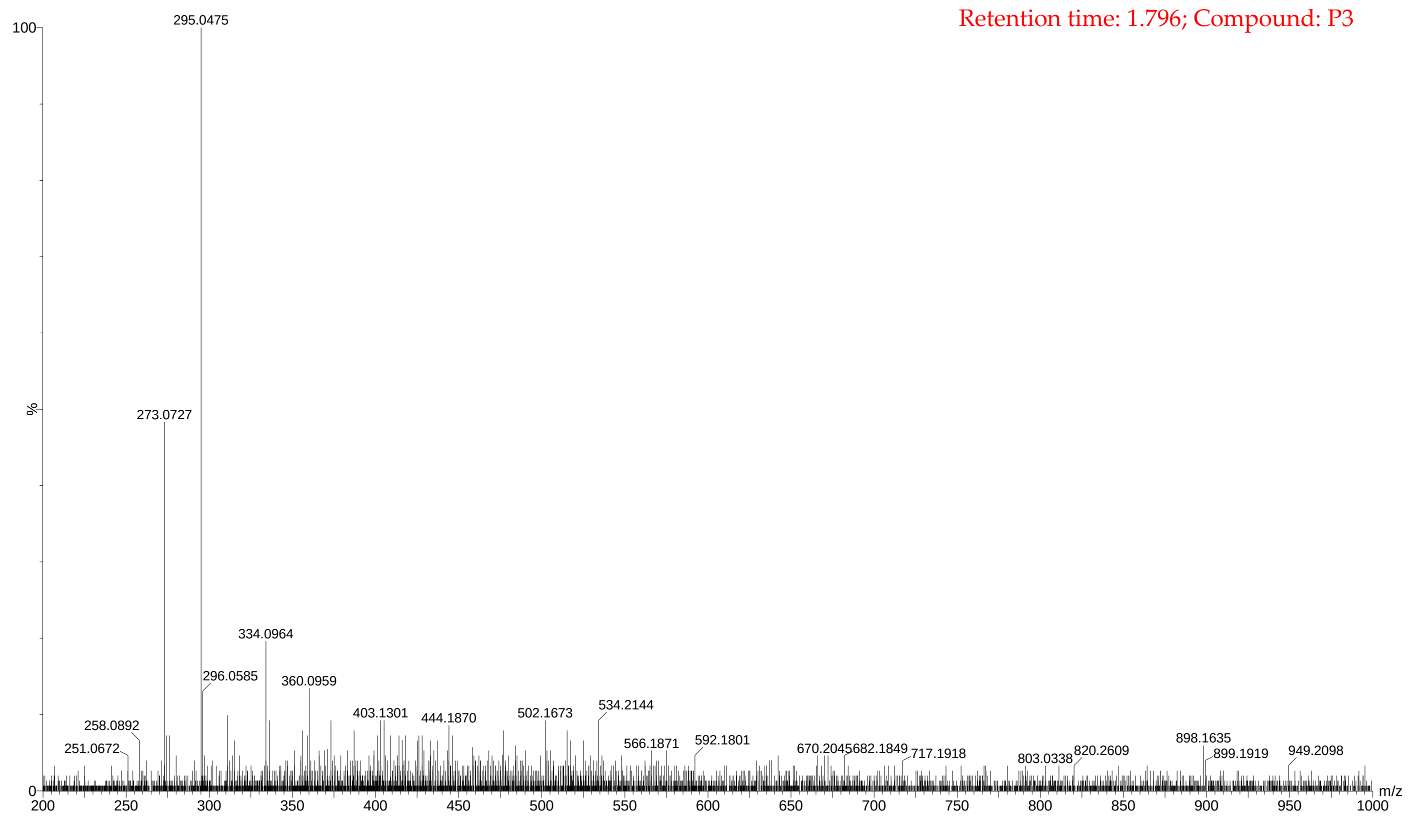

Retention time: 1.796; Compound: P3
100
%
0
295.0475
273.0727
334.0964
296.0585
360.0959
258.0892
251.0672
403.1301
444.1870
502.1673
534.2144
566.1871
592.1801
670.2045
682.1849
717.1918
803.0338
820.2609
898.1635
899.1919
949.2098
200 250 300 350 400 450 500 550 600 650 700 750 800 850 900 950 1000
m/z

(**c**)

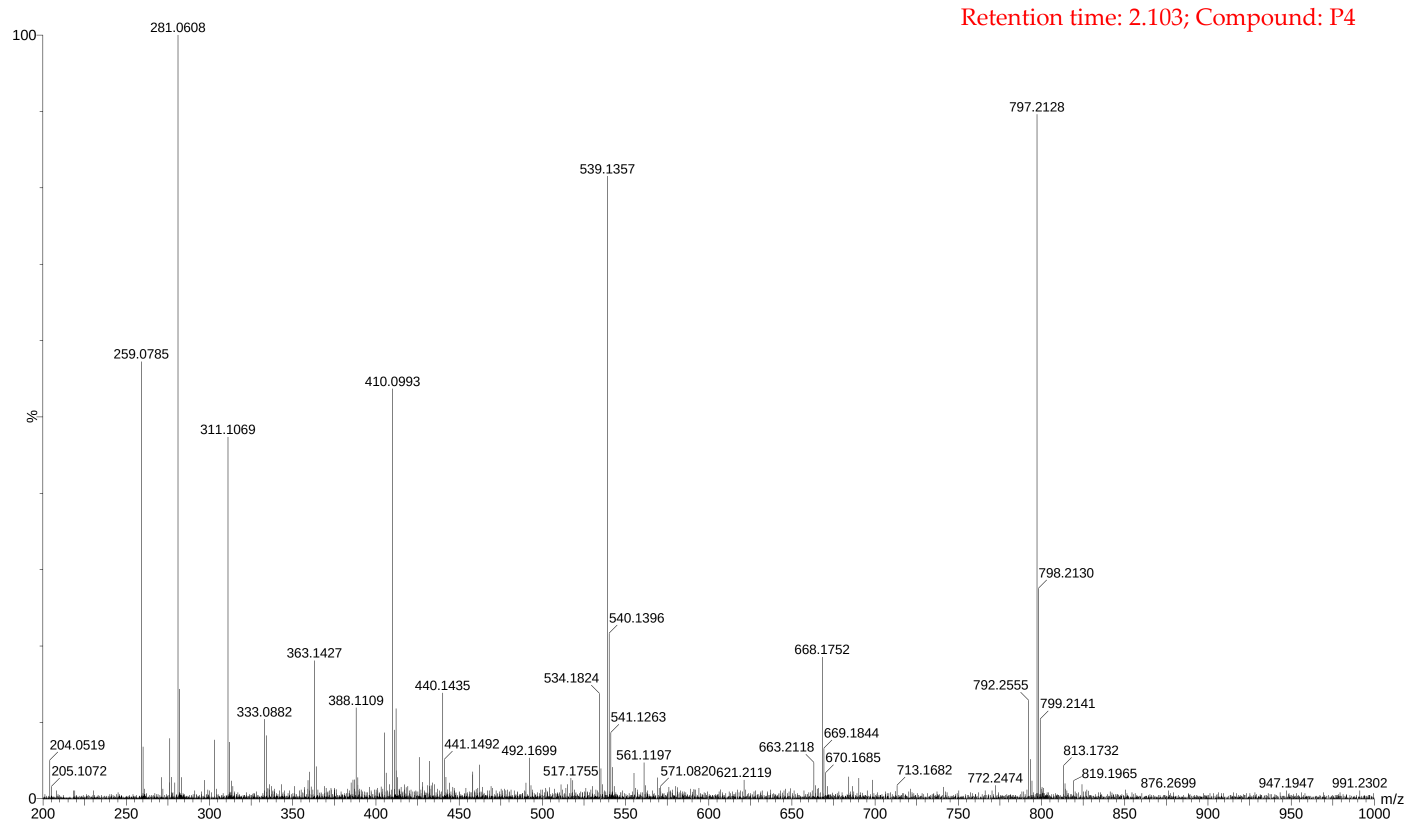

Retention time: 2.103; Compound: P4
100
%
0
281.0608
797.2128
539.1357
259.0785
410.0993
311.1069
798.2130
540.1396
668.1752
363.1427
534.1824
440.1435
792.2555
388.1109
799.2141
333.0882
541.1263
669.1844
204.0519
441.1492
492.1699
663.2118
813.1732
670.1685
561.1197
205.1072
517.1755
571.0820
621.2119
713.1682
772.2474
819.1965
876.2699
947.1947
991.2302
200 250 300 350 400 450 500 550 600 650 700 750 800 850 900 950 1000
m/z

(**d**)

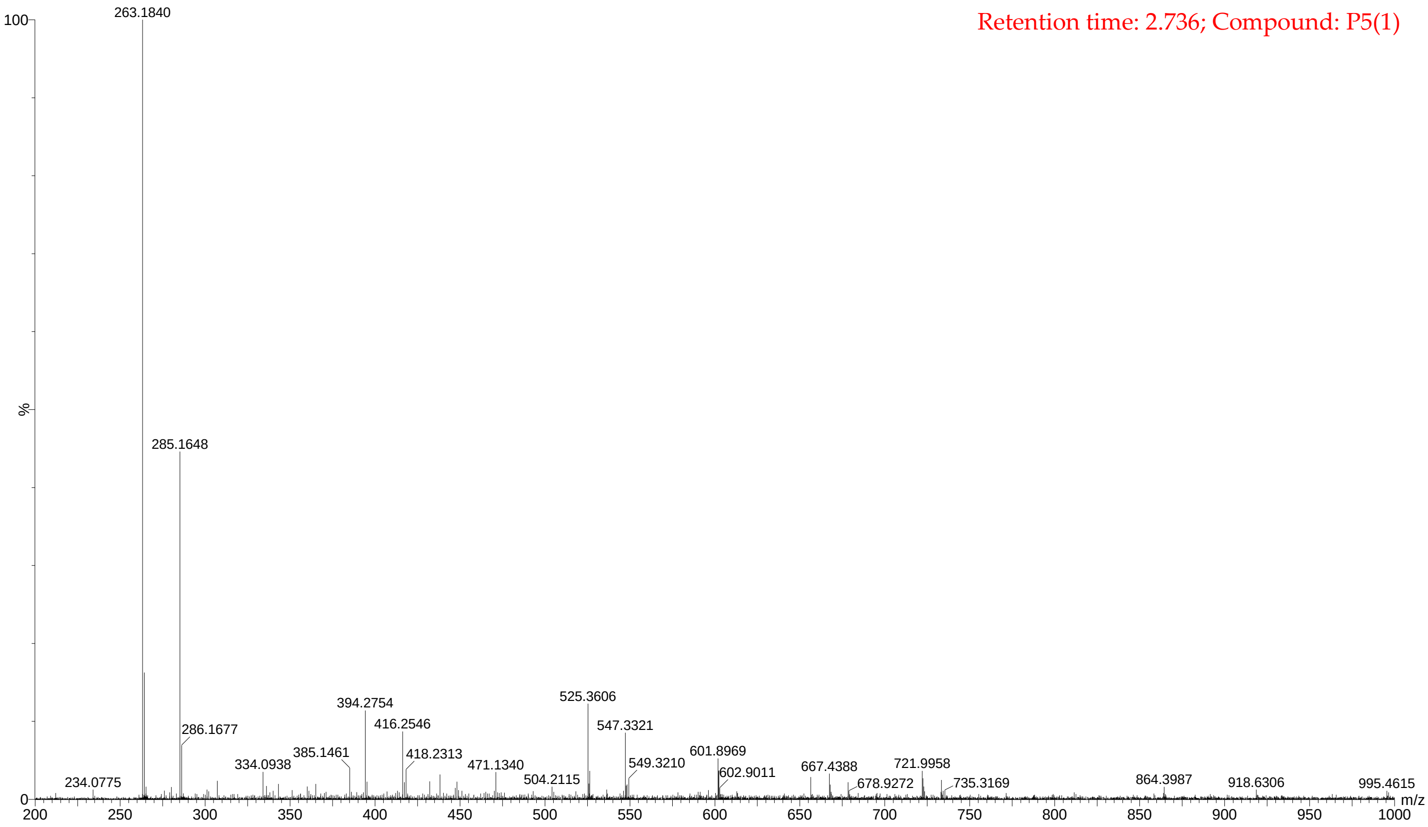
Retention time: 2.736; Compound: P5(1)
100
%
0
263.1840
285.1648
286.1677
234.0775
334.0938
385.1461
394.2754
416.2546
418.2313
471.1340
504.2115
525.3606
547.3321
549.3210
601.8969
602.9011
667.4388
678.9272
721.9958
735.3169
864.3987
918.6306
995.4615
m/z
200
250
300
350
400
450
500
550
600
650
700
750
800
850
900
950
1000

(**e**)

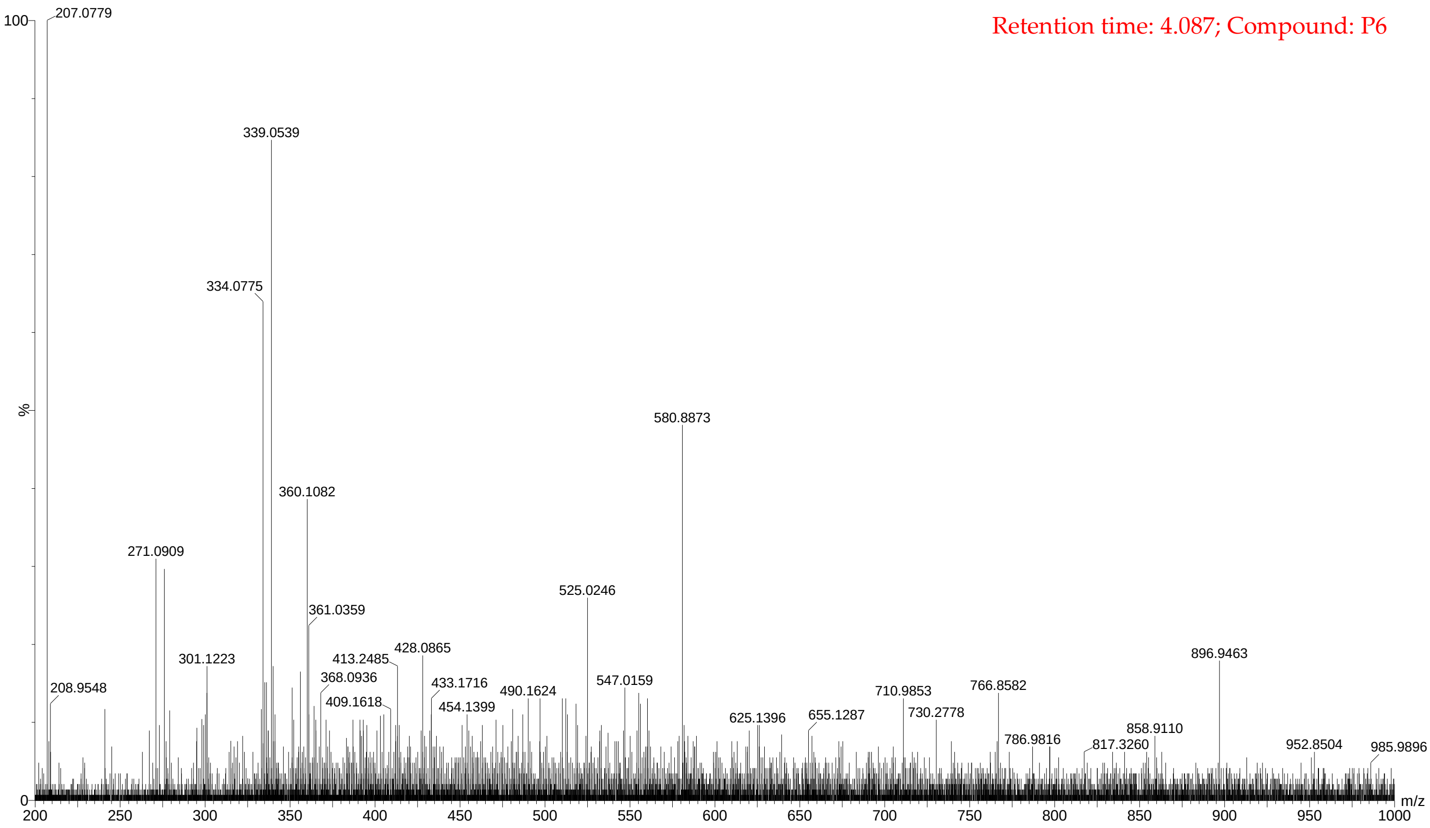
Retention time: 4.087; Compound: P6
100
%
0
207.0779
339.0539
334.0775
580.8873
360.1082
271.0909
525.0246
361.0359
428.0865
413.2485
301.1223
368.0936
208.9548
433.1716
490.1624
547.0159
409.1618
454.1399
710.9853
766.8582
896.9463
625.1396
655.1287
730.2778
786.9816
817.3260
858.9110
952.8504
985.9896
m/z
200
250
300
350
400
450
500
550
600
650
700
750
800
850
900
950
1000

(**f**)

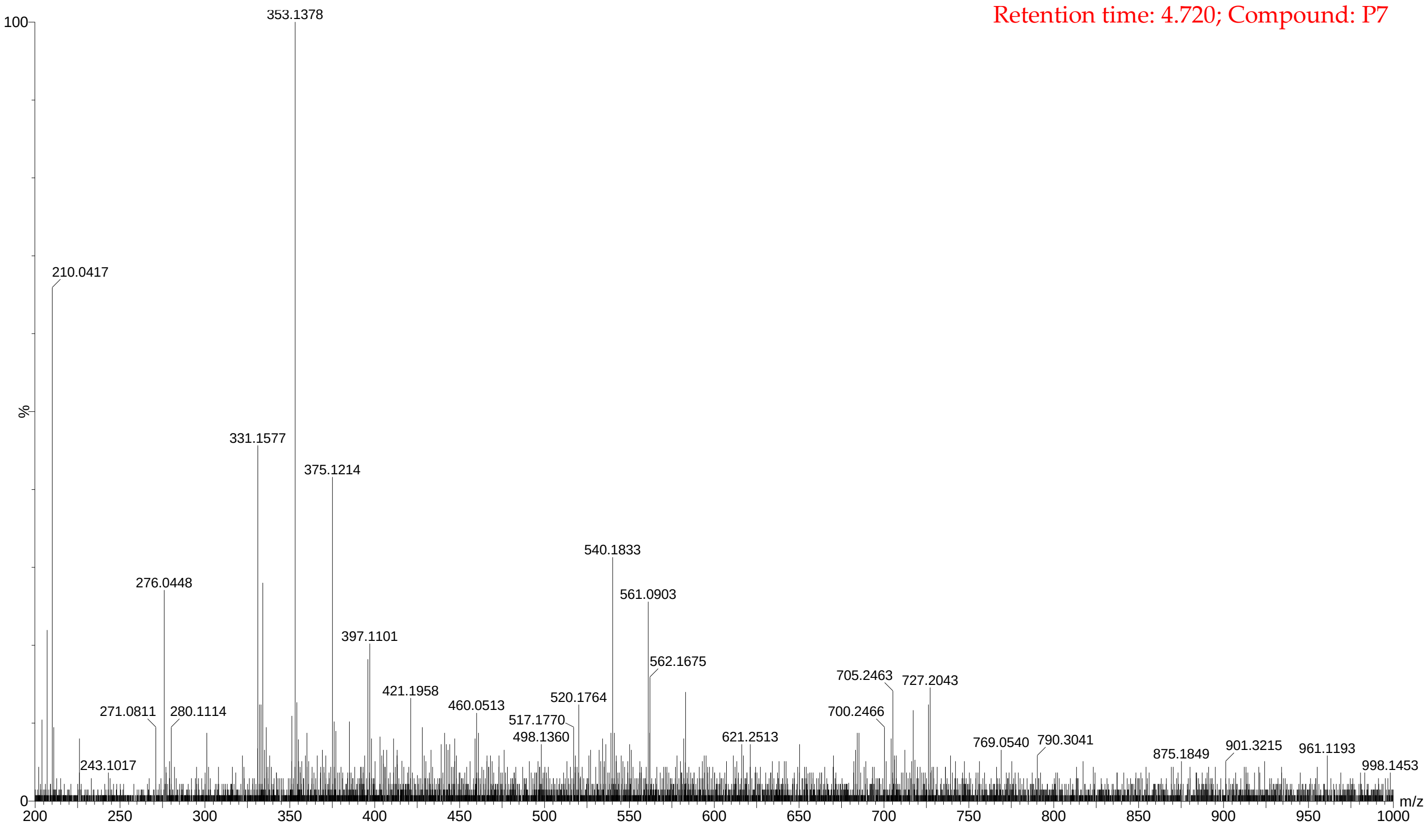

(**g**)

Figure S1. The corresponding mass spectrum data of seven peaks with retention times at 1.471 min (**a**), 1.590 min (**b**), 1.796 min (**c**), 2.103 min (**d**), 2.736 min (**e**), 4.087 min (**f**), and 4.720 min (**g**).
